# Aortic intimal sarcoma with abdominal metastasis: case report and management approach

**DOI:** 10.3389/fonc.2025.1508691

**Published:** 2025-02-12

**Authors:** Gongji Yao, Jianwei Xu

**Affiliations:** ^1^ Department of Intervention, Huzhou Central Hospital, The Fifth School of Clinical Medicine of Zhejiang Chinese Medical University, Huzhou, Zhejiang, China; ^2^ Department of Intervention, Huzhou Central Hospital, The Affiliated Central Hospital of Huzhou University, Huzhou, Zhejiang, China

**Keywords:** aortic intimal sarcoma, abdominal metastasis, MDM2, chemotherapy, PET-CT, MRI, case report

## Abstract

**Background:**

Aortic intimal sarcoma is an exceptionally rare malignancy with a poor prognosis. Tumors are predominantly located in the abdominal aorta, thoracic aorta, and thoracoabdominal aorta. Abdominal metastasis of aortic sarcoma is rarely documented, and effective treatment regimens are lacking.

**Case presentation:**

A 55-year-old female presented with recurrent abdominal pain and a history of hypertension and mesenteric thrombosis. Initial arterial computed tomography angiography (CTA) revealed multiple thrombi with significant luminal narrowing, leading to a diagnosis of aortic thrombosis. She was referred to the First Affiliated Hospital of Zhejiang University for surgical intervention. Pathological analysis confirmed a diagnosis of aortic intimal sarcoma with MDM2 positivity. One month later, the patient developed severe abdominal pain, and positron emission tomography-computed tomography (PET-CT) showed accumulation in the small intestine, jejunum, and back muscles. Palliative tumor removal was performed, and the patient received chemotherapy with platinum drugs and epirubicin. Post-treatment PET-CT indicated no significant tumor staining or progression.

**Discussion:**

Aortic intimal sarcoma is a rare neoplasm with limited treatment options. MDM2 overexpression is commonly observed, but similar histological features can appear in other conditions, making diagnosis challenging. Imaging modalities, including MRI and PET-CT, are crucial for diagnosis and monitoring. Current treatment strategies are non-standardized, but small-molecule inhibitors targeting MDM2 show promise. This case highlights the potential effectiveness of combined surgical and chemotherapeutic approaches for managing abdominal metastasis of aortic intimal sarcoma and provides a foundation for future clinical trials.

## Introduction

Aortic intimal sarcoma is an exceptionally rare aorta tumor with a grim prognosis. Predominant tumor locations include the abdominal aorta (40%), thoracic aorta (20%), and thoracoabdominal aorta (10%) (1, 2). However, abdominal metastasis of aortic sarcoma is scarcely documented. Currently, there is no effective treatment regimen available (2, 3). We present a rare case of primary aortic sarcoma with abdominal metastasis and its subsequent treatment. Herein, we report a case of primary aortic intimal sarcoma with abdominal metastasis and its treatment strategy.

## Case presentation

**Figure 1:**
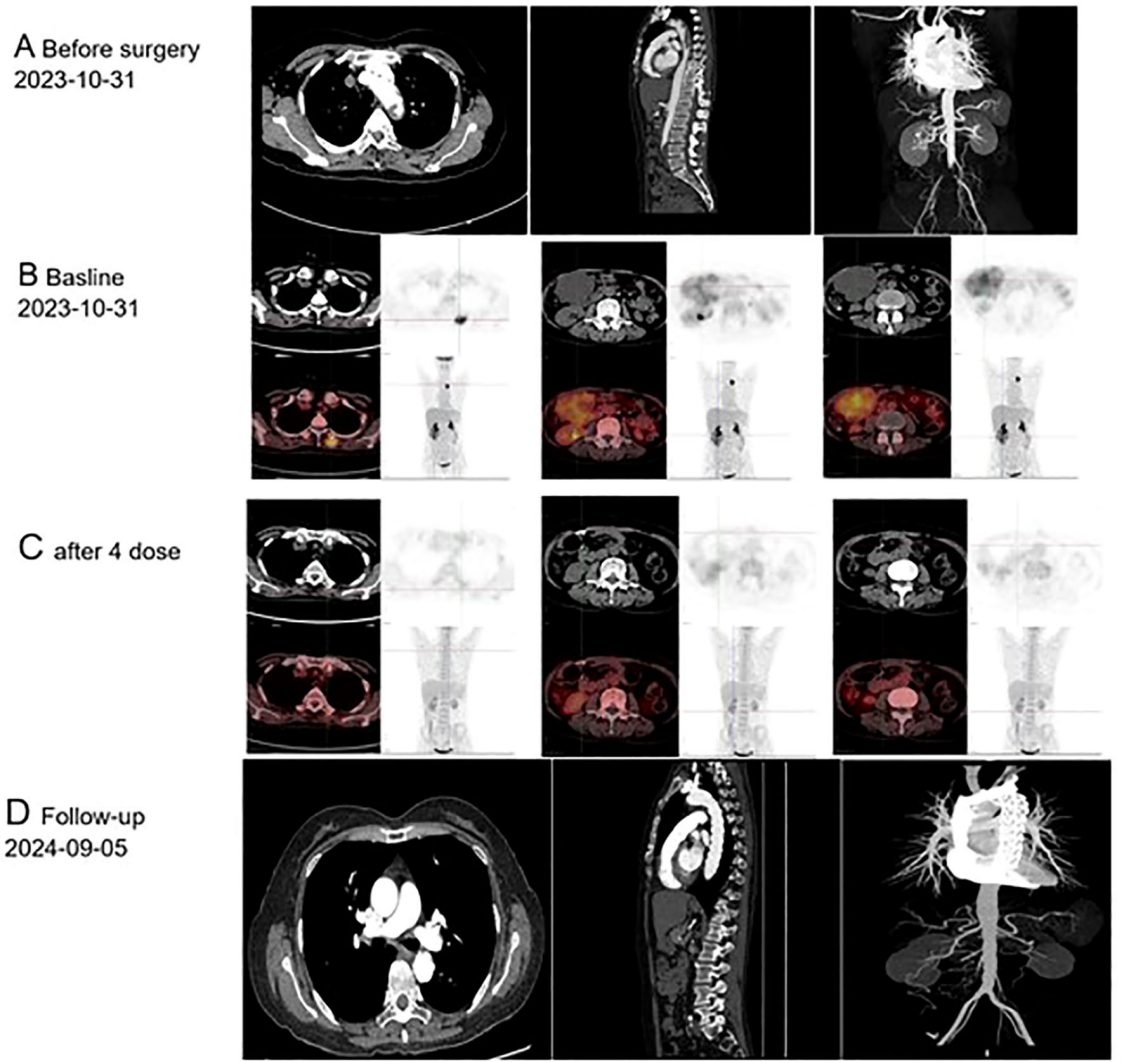
Imaging findings of primary endovascular sarcoma: **(A)** Computed Tomography conducted prior to October 31, 2023; **(B)** Positron Emission Tomography performed before December 14, 2023; **(C)** Positron Emission Tomography following four cycles of postoperative chemotherapy; **(D)** Follow-up care, September 5th, 2024.

**Figure 2:**
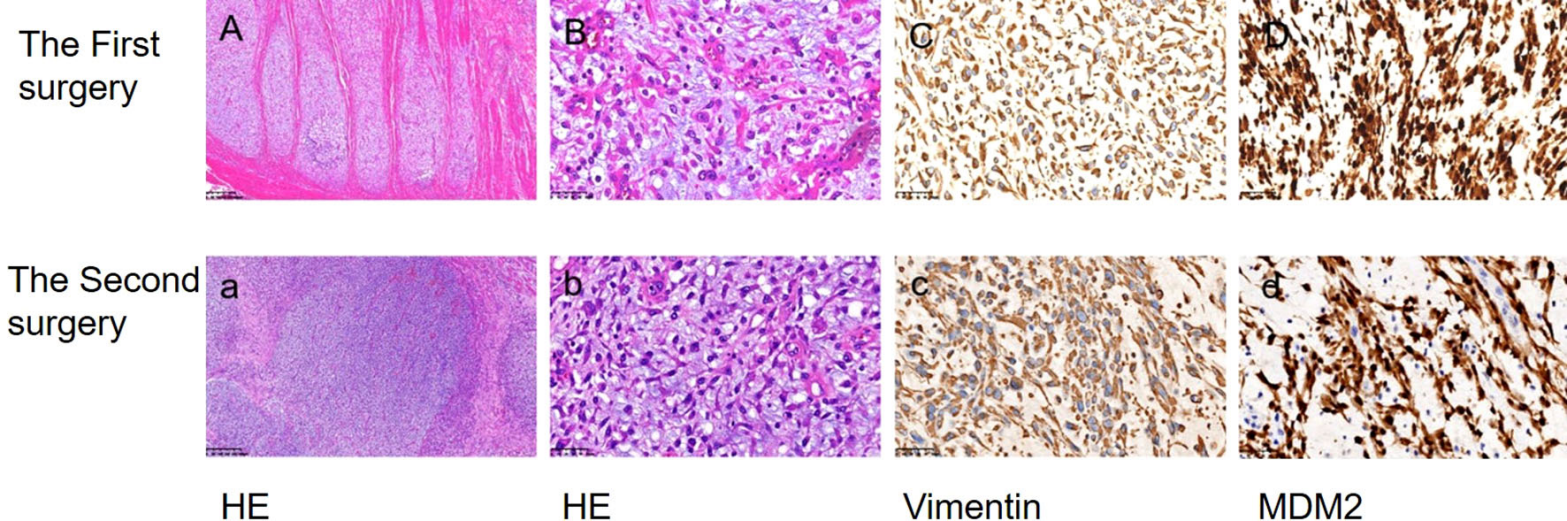
The morphological characteristics and immunohistochemical findings of biopsy specimens were examined. Hematoxylin and eosin staining (HE) revealed the presence of endometrial sarcoma cells (**A, a** ×100; **B, b** ×400). Immunohistochemical analysis demonstrated positive expression of Vimentin and MDM2 (**C, c**; **D, d** ×400).

A 55-year-old female patient was admitted to the hospital with a primary complaint of “recurrent abdominal pain for over a month, worsened for the past hour.” The patient denies smoking or alcohol consumption but has a history of hypertension and a history of mesenteric thrombosis diagnosed one month prior. Upon admission, her blood pressure was 180/110 mmHg, with all other vital signs within normal range. Arterial computed tomography angiography (CTA) revealed The aortic arch, the superior segment of the descending aorta, the inferior segment of the abdominal aorta, the bilateral iliac arteries, and the distal internal portion of the superior mesenteric artery. A filling defect is present in the balance sheet, and the local lumen exhibits severe stenosis (**Figure 1A**). The attending physician diagnosed her condition as aortic thrombosis. She was urgently transferred to the First Affiliated Hospital of Zhejiang University for surgical treatment, After meticulous surgical disinfection under general anesthesia, the patient received a longitudinal incision in the bilateral groin, and the bilateral common femoral artery was liberated. Following systemic heparinization (intravenous injection of 100U/kg heparin), the femoral artery approach catheter was utilized to access the thoracic aorta. Angiography disclosed large filling defects in the descending aorta and distal occlusion of the superior mesenteric artery. Occlusion was present from the lower abdominal aorta to the bifurcation of both iliac arteries. Once the CODA balloon was sent to the thoracic aorta along the catheter guide wire, the balloon was inflated, and the thrombus was withdrawn. Reimaging indicated that the filling defect of the thoracic aorta was reduced compared to previous imaging, and a covered stent of 26-22 *160mm was placed at the distal end of the left subclavian artery. Again, the abdominal aorta was accessed via the bilateral femoral artery approach, and a considerable amount of jelly-like neoplasms were removed through the thrombectomy catheter. The abdominal aorta, bilateral common iliac artery, and external iliac artery were found to have smooth blood flow. After the bilateral femoral artery incision was trimmed, the intima was exfoliated, and the bilateral common femoral artery incision was sutured. The femoral artery pulsated well upon opening the blood flow. The bilateral groin incisions were sutured layer by layer. Frozen section analysis identified the presence of aortic intimal sarcoma (**Figures 2A, B**), and the tumor was resected with clear margins. The tumor cells were positive for vimentin and MDM2 (**Figures 2C, D**), confirming the diagnosis of aortic intimal sarcoma.

After a month, she returned to the hospital with a recent onset of sharp, acute, and persistent abdominal pain. Positron Emission Tomography-Computed Tomography (PET-CT) showed fluorodeoxyglucose (FDG) accumulation in the small intestine, part of the jejunum, and back muscles (**Figure 1B**). The patient was transferred to the Department of Vascular Surgery for palliative tumor removal. Following general anesthesia, the patient was positioned in a supine posture and a 15-cm incision was executed in the mid-abdomen. During the surgical procedure, a palpable mass was discerned in the ascending mesocolon (approximately 10*10 cm), which permeated the mesocolon and invaded the jejunum. 40 cm distant from the Treitz ligament, several enlarged lymph nodes were detectable in the mesentery, concurrent with adhesion of the omentum and small intestine. Moreover, 5.0*5.0 masses were identified subcutaneously on the back. In light of the intraoperative observations, resection of the abdominal and back masses, local resection of the small intestine, jejune-jejunal side-to-side anastomosis, and lysis of intestinal adhesions were carried out. Histopathological examination confirmed intimal sarcoma, Weakly positive for vimentin, staining positive for MDM2 (**Figures 2C, D**). Two weeks after surgery, the patient received chemotherapy with Cisplatin (38 mg, Days 1-3) combined with epirubicin (110 mg, Day 1) every three weeks without radiation treatment. After four cycles of this regimen, the patient’s symptoms were completely resolved with no adverse events. PET-CT revealed No fluorodeoxyglucose accumulation (FDG) was observed in the operative area (**Figure 1C**). The patient has exhibited no tumor progression and has been under our follow-up care from October 1, 2023, to September 20, 2024 (**Figure 1D**). So we think The local surgical treatment combined with chemotherapy may represent an effective approach for peritoneal metastasis of primary aortic intimal sarcoma. However, further cases are required to substantiate this conclusion.

## Discussion

Aortic intimal sarcoma is an exceptionally rare neoplasm, and due to the rarity and limited clinical data available on this disorder, the majority of patients are diagnosed at advanced stages, resulting in a poor prognosis. The median survival for affected individuals is approximately 16 months (range: 0–168 months) (1, 2), with 3-year and 5-year survival rates at approximately 11.2% and 8%, respectively. The male to female ratio among patients is about 2:1, with the average age at diagnosis being 60 years (range: 48–85 years) (2). The principal clinical manifestations include vascular embolism or occlusion, primarily caused by arterial emboli.

Here, we report a case of aortic sarcoma in a 55-year-old female patient with abdominal metastasis, presenting with severe abdominal pain and symptoms of arterial embolization, which led to an initial misdiagnosis. During the patient’s first surgery, pathological examination revealed MDM2 positivity. The diagnosis of primary aortic sarcoma was established based on its location, pathological findings, and medical history. Approximately one month after surgery, the patient experienced a recurrence of severe abdominal pain. PET-CT scans were conducted on the small intestine and back muscles, followed by resective procedures in these areas and chemotherapy administration two weeks later.

Histologically, MDM2 overexpression is noted in most cases; however, similar histological features can occur in other conditions that are not specific to this diagnosis. Therefore, the diagnosis primarily relies on correlating clinical manifestations with imaging findings (4). Vimentins are class-III intermediate filaments found in various non-epithelial cells, especially mesenchymal cells. vimentin as an exclusive marker on sarcoma circulating tumor cells regardless of the tissue origin of the sarcoma (5). In Burke and Virmani’s analysis of nine cases of aortic intimal sarcoma, vimentin expression was positive (6). Therefore, we hold the belief that the main immune characteristics of aortic endovascular sarcoma are as follows: 1. Vimentins derived from the interstitial tissue should be positive; 2. Endometrial sarcomas typically exhibit poor differentiation and nonspecific characteristics, with MDM2 expression observed in over 70% of cases (7). To more accurately differentiate types of sarcomas, it is essential to conduct smooth muscle staining and epithelial marker staining. These procedures are indeed necessary when positive MDM2 is identified, as they provide critical diagnostic information.

Some studies suggest that magnetic resonance angiography using gadolinium is the most sensitive imaging modality. Magnetic resonance imaging is crucial as tumors often resemble atherosclerotic plaques on CT imaging, making differentiation difficult (2, 8). In previous studies, PET-CT was primarily used in cases with a high suspicion of early metastasis. In pulmonary endothelial sarcoma, FDG-PET imaging typically demonstrates high F-18 uptake, with a reported mean maximum standardized uptake value (SUVmax) of 7.63 ± 2.21 (9, 10). Another study established a cutoff value of 3.5 for SUVmax to differentiate between cardiac malignancies and benign tumors (11). Wittram et al. observed that the SUVmax values for pulmonary embolism ranged from 0.45 to 3.03 (12). It has been reported that single PET-CT findings are insufficient to definitively differentiate between endometrial sarcoma and pulmonary embolism (13)]. Consequently, the interpretation of PET/CT results should be integrated with additional relevant clinical and imaging information for a comprehensive evaluation. Based on the following,we thinkThis imaging model PET-CT may be useful in: (1) estimating disease stage and tumor burden; (2) Postoperative follow-up to evaluate the residual tumor; (3) Evaluation of early response to neoadjuvant and adjuvant therapy (9).

Given the rarity of aortic sarcoma, no standardized treatment protocols currently exist (2). In the instance of localized disease with no evidence of metastasis, radical resection seems to be the most efficacious treatment option (14). In pulmonary endometrial stromal sarcomas, both doxorubicin and ifosfamide combination chemotherapy regimens have demonstrated efficacy, contingent upon the histological subtype of these tumors (15). Other drugs like carboplatin, epirubicin, cyclophosphamide, gemcitabine, dacarbazine, eposide, and vinorelbine have also demonstrated a positive effect in some cases of pulmonary endometrial stromal sarcomas (16). The patient had a history of hypertension and had undergone two surgical procedures, resulting in a significant cardiac burden. To mitigate this burden, Epirubicinand cisplatin was selected due to its lower cardiotoxicity.

Due to its overexpression of MDM2, small-molecule inhibitors targeting MDM2 may be a potential treatment option. In a recent open-label phase 1b/2 study of MDM2 inhibitors, 20% of patients showed sustained survival for over 15 months (17). However, this study was limited to only 10 cases and had several limitations, so it cannot definitively establish the efficacy of targeted therapy. We present a case of intimal sarcoma with abdominal metastasis treated through a combination of surgical intervention and postoperative chemotherapy. While it is difficult to exclude potential synergistic effects from this combined treatment on favorable outcomes, these findings provide a foundation for clinical trials exploring therapies for Aortic intimal sarcoma.

## Data Availability

The raw data supporting the conclusions of this article will be made available by the authors, without undue reservation.
